# Implementing the Routine Use of Electronic Mental Health Screening for Youth in Primary Care: Systematic Review

**DOI:** 10.2196/30479

**Published:** 2021-11-19

**Authors:** Rhiannon Martel, Matthew Shepherd, Felicity Goodyear-Smith

**Affiliations:** 1 Department of General Practice & Primary Health Care Faculty of Medical & Health Science University of Auckland Auckland New Zealand; 2 School of Psychology Massey University Auckland New Zealand

**Keywords:** adolescent, mental health, risk behavior, screening, primary care

## Abstract

**Background:**

Adolescents often present at primary care clinics with nonspecific physical symptoms when, in fact, they have at least 1 mental health or risk behavior (psychosocial) issue with which they would like help but do not disclose to their care provider. Despite global recommendations, over 50% of youths are not screened for mental health and risk behavior issues in primary care.

**Objective:**

This review aimed to examine the implementation, acceptability, feasibility, benefits, and barriers of e-screening tools for mental health and risk behaviors among youth in primary care settings.

**Methods:**

Electronic databases—MEDLINE, CINAHL, Scopus, and the Cochrane Database of Systematic Reviews—were searched for studies on the routine screening of youth in primary care settings. Screening tools needed to be electronic and screen for at least 1 mental health or risk behavior issue. A total of 11 studies that were reported in 12 articles, of which all were from high-income countries, were reviewed.

**Results:**

e-Screening was largely proven to be feasible and acceptable to youth and their primary care providers. Preconsultation e-screening facilitated discussions about sensitive issues and increased disclosure by youth. However, barriers such as the lack of time, training, and discomfort in raising sensitive issues with youth continued to be reported.

**Conclusions:**

To implement e-screening, clinicians need to change their behaviors, and e-screening processes must become normalized into their workflows. Co-designing and tailoring screening implementation frameworks to meet the needs of specific contexts may be required to ensure that clinicians overcome initial resistances and perceived barriers and adopt the required processes in their work.

## Introduction

More than 90% of New Zealand secondary school students visit a primary care provider, such as a family physician or primary care nurse, at least once per year [1]. Adolescents often present at primary care clinics with nonspecific physical symptoms when, in fact, they have at least 1 mental health or risk behavior (psychosocial) issue with which they would like help but do not disclose to their care provider [2-4]. Incidence rates of youth psychosocial issues are higher for New Zealand’s indigenous Māori population, whose access to appropriate care is less than that of the general New Zealand population [5]. Mental health issues generally include anxiety and depression but may also include more general distress resulting from a variety of stressors and difficulty with controlling anger. Risk behaviors include substance misuse (nicotine, alcohol, and recreational drugs), eating and conduct distress, sexual health, physical inactivity, and exposure to abuse and problem gambling or gaming. A full psychosocial assessment can help with identifying these concerns, but the young person must be willing to discuss personal and delicate issues, sometimes with someone they do not know [6]. Screening can reveal issues that could otherwise be overlooked and can facilitate discussions about psychosocial concerns between care providers and youth [7-9].

Currently, year 9 students (aged 13-14 years) in New Zealand decile 1 to decile 3 secondary schools undergo a routine psychosocial assessment that uses the Home, Education/Employment, Eating, Activities, Drugs and Alcohol, Sexuality, Suicide/Depression, and Safety (HEEADSSS) assessment tool [10]. This is a multi-item, interview-based assessment tool that is also used by some clinicians in primary health care during consultations with adolescents. Although the HEEADSSS tool is used nationally and internationally, it is not validated, it can be time-consuming to use (sometimes taking up to 2 hours to complete), and the results are variable [11,12].

A number of screening tools are available for youth psychosocial issues, but most cover a single domain [13], and administering and interpreting these tools can be time-consuming [14]. Primary care clinicians may be uncertain about which screening tools are suitable for use in certain clinical contexts. Many tools rely on care providers having the skills, knowledge, expertise, and experience to initiate the screen, interpret the results, and provide appropriate interventions [7]. Care providers often describe being underresourced in terms of time, the availability of appropriate tools, training, and their experience in youth health [15]. Care providers have also cited a lack of awareness of appropriate agencies and available support services as a further barrier to screening [3,7,15,16].

Underpinned by national and international policies and strategies, global recommendations state that young people who seek help from their care providers should be routinely screened for psychosocial issues [17]. Despite this, such screening occurs in less than 50% of primary care consultations with youth, meaning that over half of adolescent mental health concerns go undetected [7,8].

The aim of this literature review was to examine the implementation of e-screening tools for psychosocial issues among youth in primary care settings. Specifically, we aimed to determine whether e-screening has been performed opportunistically or systematically, whether such screening has targeted those who were deemed at risk for mental health or risk behavior (psychosocial) issues, whether e-screening has been conducted in the waiting room prior to consultation or at another time, and whether e-screening has been initiated by an administrator (either a research assistant or a clinic staff member). The objectives were to explore different conditions and settings where routine e-screening for youth psychosocial issues is undertaken and to identify the perceived acceptability and benefits of, barriers to, and feasibility of the implementation of such screening.

## Methods

### Search Strategy

The search strategy was devised through discussion with a specialist librarian and all review authors. The electronic databases MEDLINE, CINAHL, Scopus, and the Cochrane Database of Systematic Reviews were searched for studies on screening for mental health issues and risky behaviors among youth. The search was conducted by using search strings that incorporated wildcard symbols (Multimedia Appendix 1). Search results were exported to bibliography software and recorded in a PRISMA (Preferred Reporting Items for Systematic Reviews and Meta-Analyses) diagram.

### Inclusion and Exclusion Criteria

All research studies published in English that conducted the e-screening of psychosocial issues among young people up to May 2020 were included. There was no publication date range for excluding studies. The inclusion criteria included studies involving the e-screening of youth in primary care. e-Screening involved the use of web-based screening tools that were delivered by a mobile device, an e-tablet, a computer, or another digital device. Youth were defined as young people aged between 12 and 25 years. Primary care settings were community-based health settings that catered to either all patients (general practice or family health services) or youth specifically (school-based clinics or youth clinics). The inclusion criteria included studies that addressed facilitators and barriers to and the process, implementation, and feasibility of using e-screening tools in primary care. The exclusion criteria were study protocols (no data available) and studies in which screening was not conducted on young people, screening was not for psychosocial issues, or screening was not the focus of the research. Studies were also excluded if the screen was not electronic or was not conducted in a primary care setting. Non-English papers were excluded.

### Screening

Titles were screened for initial eligibility, and duplicates were removed by using bibliography software. Abstracts were independently screened by 2 authors and cross-checked for agreement. The included abstracts were reviewed and further excluded if they did not meet the eligibility criteria. Afterward, the full papers of included studies and further studies identified through hand searching were reviewed, and those that did not meet the eligibility criteria were excluded. A second researcher checked that the full-text papers were eligible for inclusion.

### Analyses

The items to be coded from the included papers were decided upon via discussion among the research team members. The studies were classified based on the country of origin, study design, type of data, clinical setting, people who were selected as participants (eg, age range), and people who had recruited them (eg, the research assistant of a clinical staff member). The lead author tabulated the specific tools and screening domains, along with any additional tools that were used, the time and duration of screening, and the place in which screening occurred. Data on the types of measures used (eg, utility, acceptability, feasibility) and the analyses undertaken were extracted and synthesized from the studies. The study quality was assessed by identifying potential biases, limitations, and strengths. FGS reviewed the process at various stages, as well as the included papers and tables, and provided feedback. Due to the heterogeneity of the studies, a meta-analysis was not possible.

## Results

### Identification and Screening of Studies

**Figure 1:**
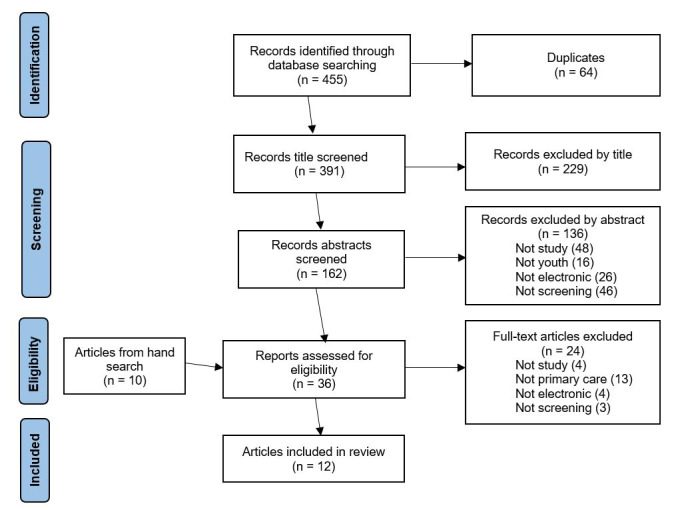
PRISMA flow diagram.

A total of 455 articles were identified, and after the screening and hand-searching processes, 12 articles reporting 11 studies were included in the review (Figure 1).

### Study Characteristics

The included papers described 11 studies that were conducted between 2009 and 2018 [6,16,18-27]. The designs used in the reviewed studies were a case study [16], co-design and descriptive studies [19,22], a translational study [20], quasi-experimental studies [18,21,23,24], and randomized trials [25-27]. All studies included quantitative data, and 4 were mixed methods studies [16,18,23]. All studies were carried out in high-income countries, and nearly half (5/11, 45%) were conducted in family health clinics, general practice clinics, or primary care clinics. Study sites also included pediatric primary care clinics, an integrated health clinic, school clinics, and a colocated youth clinic. Most of the studies (8/11, 73%) recruited both young people and care providers as participants. Youth were recruited from clinic waiting rooms when they attended their routine medical reviews, while care providers were recruited from participating clinics. In one study conducted in New Zealand, the youth participants were mostly indigenous Māori [19].

All sixth- to 12th-grade students at a public school were eligible to participate in 1 project, and one study did not recruit young people per se but used deidentified data from electronic medical records. Youth participants’ ages ranged from 11 to 25 years across all of the studies (Table 1).

**Table 1:** Study designs, participants, and settings.

Study authors	Country	Study design	Data type	Setting	Participant selection criteria
Bilardi et al [18]	Australia	Quasi-experimental	Mixed	Family health clinic	All 16- to 24-year-olds attending their annual reviews (N=871) and primary care providers
Bradford and Rickwood [6]	Australia	Quasi-experimental	Quantitative	Youth clinic	12- to 25-year-olds (n=339) and 13 clinicians
Curtis et al [20]	United States of America	Translational	Quantitative	School clinic	All sixth- to 12th-grade pupils from 1 school (N=248)
Diamond et al [22]	United States of America	Descriptive	Quantitative	Family health clinic	12- to 21-year-olds in primary health care waiting rooms (N=415)
Gadomski et al [23]	United States of America	Quasi-experimental	Mixed	Urban and rural clinics	Consecutive patients aged <18 years attending their annual reviews (N=72) and primary care providers
Goodyear-Smith et al [19]	New Zealand	Co-design	Mixed	Youth clinic	Consecutive patients aged 12-24 years (N=30) and care providers
Harris and Knight [21]	United States of American and Czech Republic	Quasi-experimental	Quantitative	Family health clinic	All patients aged 12-18 years undergoing routine care (United States of America: n=2106; Czech Republic: n=589)
Olson et al [24]	United States of America	Quasi-experimental	Quantitative	Primary care clinic	Consecutive patients aged 11-19 years (N=1052) and primary care providers
Riese et al [25]	United States of America	Randomized controlled trial	Quantitative	Pediatric primary care clinic	13- to 19-year-olds (n=120) and primary care providers (n=14)
Sterling et al [27]	United States of America	Randomized controlled trial	Quantitative	Integrated health clinic	Primary care providers caring for ≥50 eligible youths (N=52; EMR^a^ data on 1871 youths were analyzed)
Webb et al [16]	Australia	Case study	Mixed	General practice clinic	14- to 25-year-olds (n=87), general practitioners (n=4), and support staff (n=10)

^a^EMR: electronic medical record.

### Initiation and Completion of Screening

In a majority of studies (7/11, 64%), e-screening was initiated by a research assistant before a young person’s consultation with their clinician (Table 2). In one study, young people were given the details of a web-based tool at the end of their consultation by either a clinician or a clinic administration staff member. Youth participants were invited to access and complete the e-screen either before leaving the clinic or later at home, but completion rates were low [18]. Clinic administration staff initiated the screen in 2 of the studies, and in a school-based project, a guidance counselor initiated it. Young people completed the screen on a mobile device; most did so in the waiting room preconsultation. Once the screen was completed, the results were immediately available to the care provider.

A variety of screening tools were used in the studies reviewed, of which some (4/11, 36%) were validated. The majority of the tools were multi-item tools, and all but one study [18] included screening for alcohol and drugs. Screens that only covered 1 domain were used in 3 studies—2 studied substance abuse screening and 1 studied sexual health risk assessment (Table 2).

**Table 2:** Screening tools, domains, screen validation, the location and duration of screens, and screen initiators.

Study authors	Tool	Domains screened	Links	Screening time (location)	Screening duration	Screen initiator
Bilardi et al [18]	Check Your Risk	Sexual health	—^a^	Postconsultation (clinic or home)	—	Youth
Bradford and Rickwood [6]	My Assessment	Home, education, eating, activities, alcohol or drug use, tobacco, sexual health, emotions, and safety	—	Preconsultation (clinic)	10-15 minutes	Research assistant
Curtis et al [20]	CRAFFT^b^ instrument (validated)	Alcohol and drugs	Alcohol and drug information	Preconsultation (school clinic)	15 minutes	School counselor
Diamond et al [22]	BHS^c^ (validated)	Medical, family, school, safety, sexuality, abuse, nutrition, eating, anxiety, trauma, depression, alcohol or drug use, suicidality, and psychosis	BDI-II^d^, MSSI^e^, and TSC^f^	Preconsultation (waiting room)	8-12 minutes	Research assistant
Gadomski et al [23]	DartScreen	Nutrition, exercise, alcohol or drug use, school, mental health, depression and anxiety, and sexual health	PHQ^g^, GAD-2^h^, and SBQ^i^	Preconsultation (waiting room)	9.5 minutes	Research assistant
Goodyear-Smith et al [19]	YouthCHAT (validated)	Smoking, alcohol or drug use, gambling, eating disorder, depression, anxiety, stress, sexual health, abuse, conduct, anger, and inactivity	PHQ-A^j^, GAD-7^k^, SACS^l^, and ASSIST^m^	Preconsultation (waiting room)	—	Research assistant
Harris and Knight [21]	CRAFFT instrument (validated)	Alcohol or drug use	CRAFFT instrument	Preconsultation	5 minutes	Research assistant
Olson et al [24]	Based on GAPSQ^n^	Family, medical, safety, smoking, sexuality, activity, mental health, body image, school, relationships, nutrition, conduct	Alcohol and drug information	Preconsultation (clinic)	9-11 minutes	Admin staff
Riese et al [25]	TickiT (with and without the YRBS^o^)	Home, education, eating, activities, alcohol or drug use tobacco, sexual health, emotions, safety	Selected YRBS	Preconsultation (waiting room)	8.4 minutes	Research assistant
Sterling et al [26,27]	TWCQ^p^	Alcohol or drug use, mood, and suicidality	CRAFFT instrument	Preconsultation (clinic)	—	Admin staff
Webb et al [16]	Check Up general practitioner app	Home, education, eating, activities, alcohol or drug use, tobacco, sexual health, emotions, and safety	—	Preconsultation (general practice clinic)	10-14 minutes	Research assistant

^a^Not applicable.

^b^CRAFFT: Car, Relax, Alone, Forget, Friends, Trouble.

^c^BHS: Behavioral Health Screen.

^d^BDI-II: Beck Depression Inventory-II.

^e^MSSI: Modified Scale for Suicidal Ideation.

^f^TSC: Trauma Symptom Checklist.

^g^PHQ: Patient Health Questionnaire.

^h^GAD-2: Generalized Anxiety Disorder 2-item.

^i^SBQ: Suicide Behavior Questionnaire.

^j^PHQ-A: Patient Health Questionnaire-Adolescent Version.

^k^GAD-7: Generalized Anxiety Disorder 7-item.

^l^SACS: Substances and Choices Scale.

^m^ASSIST: Alcohol, Smoking and Substance Involvement Screening Test.

^n^GAPSQ: Guidelines for Adolescent Preventive Services Questionnaire.

^o^YRBS: Youth Risk Behavior Survey.

^p^TWCQ: Teen Well Check Questionnaire.

### Implementation Factors Included in the Studies

The acceptability and utility of e-screening tools for both care providers and young people were outcomes that were measured in 5 of the studies, and 8 studies described the impact that reviewing the results of a screen had on discussions and engagement during the postscreen consultation (Table 3). Two studies evaluated whether training care providers, providing them with resources, and obtaining support from other clinicians had any influence on rates of the psychosocial e-screening of youth. Another analyzed screening rates after the implementation of a computer-based, self-reported, previsit screen for youth psychosocial issues.

**Table 3:** Sources of data, study measures, potential biases, limitations, and strengths.

Study authors	Data sources	Measures	Analysis	Bias	Limitations	Strengths
Bilardi et al [18]	EMR^a^ data, and interviews	Number of tests at 6 months pre- and postintervention, youth feedback, and barriers to use	2-sided *P* values, descriptive statistics, and thematic analysis	Training increases screening awareness	Small sample and no feedback	Real clinical situation
Bradford and Rickwood [6]	My Assessment data and questionnaires	Acceptability, feasibility, utility, reported behaviors, and barriers to use	Descriptive statistics and the comparison of control and intervention psychometrics	Missing data	Single center	Large sample size, a response rate of 87%, and a quasi-experimental design
Curtis et al [20]	EMR data	Utility in school, screening and detection rates, counseling acceptability, sustainability barriers, and barriers to use	Formative evaluation	Bias toward financially stable families	No usage data	Tested in school
Diamond et al [22]	Survey	Utility and acceptability, screen understandability, honest disclosure, and barriers to use	Descriptive statistics and odds ratios	Researcher-created tool	Nonrandom sample	Identifies barriers
Gadomski et al [23]	Interviews, audio recordings, and a youth survey	Information provided, question types, brief intervention delivery rates, engagement, and issues addressed	Inductive thematic approach	Effect of recording	Nonrandom sample	Real clinical situation
Goodyear-Smith et al [19]	Surveys, focus groups, and interviews	Assessment utility, youth and care provider acceptability, and barriers to use	Descriptive statistics and thematic analysis	Nonrepresentative sample	Small sample and no control	Real clinical situation
Harris and Knight [21]	Postvisit survey and EMR data	Advice-to-quit rates, likelihood of following advice, youth satisfaction, responses to the 3- and 6-month postscreen survey, and barriers to use	Chi-square tests (categorical data), *t* tests (continuous data), and longitudinal data	Self-reported data (potential recall error and the social desirability effect)	Nonrandomized study and small sample	Consistent with previous study
Olson et al [24]	Exit surveys	Youth satisfaction, youths’ perceptions of care provider attention and discussions, and barriers to use	Chi-square and Fisher exact tests	Sample mostly consisting of White, middle-class participants	Small study	—^b^
Riese et al [25]	Exit survey	Care providers’ impressions of the utility of disclosures and discussions and barriers to use	Descriptive statistics	Specific setting and population	Small sample	Cluster-randomized study
Sterling et al [27]	EMR data	Effect on screening rates, effect of adding BHCs^c^ (initiation and engagement with and without a BHC), and barriers to use	Descriptive statistics and bivariate and logistic models	Integrated clinics	Established EMR	Diverse population
Webb et al [16]	Focus groups, interviews, and utility measures	Rates of use, barriers and facilitators, and the feasibility of use	Descriptive statistics and thematic analysis	Socioeconomically advantaged population	Single case study	—

^a^EMR: electronic medical record.

^b^Not available.

^c^BHC: behavioral health clinician.

Data were gathered by using a range of methods. Acceptability and feasibility data were gathered via questionnaires, focus groups with young people, interviews with clinic staff, and exit surveys. Transcripts of audio recordings of consultations, focus groups, and free text in surveys were used to obtain qualitative data on the effect that screens had on consultations. Deidentified pre- and postintervention data from electronic health records, Likert-style survey questions, and yes-no survey questions provided quantitative data. Further information about the study measures, potential biases, limitations, and strengths of each study are summarized in Table 3.

### Findings of Studies

#### Summary of Studies’ Findings

None of the reviewed studies had changes in screening rates as the main focus. Nonetheless, offering access to a web-based screening tool increased screening rates in all of the studies except one, in which access to the screening tool was provided at the end of the consult. In this study, care providers often forgot to give the link or only gave it to youth who they perceived to be at high risk [18]. When the screen covered several domains, multiple risk behaviors were disclosed by over one-third of young people.

#### Preintervention Training

Preintervention education was available to participating care providers in 7 studies and was not discussed in the remainder of the studies. Care providers in one study received no formal training, although they were supplied with printed instructional materials on the guidelines for the screening, management, and treatment of chlamydia. In other studies, the research team trained staff to use the screening tool and offered support and resources to guide the delivery of brief interventions. When care providers attended 2 or more of these education sessions, the likelihood of e-screening for psychosocial issues taking place and brief interventions being delivered increased.

#### Barriers to Using Web-Based Screening Tools

Despite the considerable heterogeneity of these studies, commentaries on barriers to use were successfully extracted from all but one study. Barriers, which were identified by young people, to using web-based screening tools were only mentioned in 1 study. In this study, youth perceived a lack of privacy when completing the screen in the waiting room [25]. However, all but one study [23] identified barriers preventing care providers from routinely e-screening youth for psychosocial issues. The cited barriers included a lack of time, knowledge, training, and awareness of referral options [18,24,25,28]. Some care providers were uncomfortable with raising sensitive issues with young people, as they were concerned that youth might be too embarrassed or worried about confidentiality to discuss psychosocial issues with them [18,20]. Additionally, a lack of staff and high staff turnover [24,28] resulted in a barrier to screening, and in one study, staff were worried that technology could impair face-to-face engagement with young people [19].

#### Effect on Consultation

In two studies, care providers found that they were able to include e-screening and brief counseling into the time allocated for standard consultations [25,26]. Following the completion of a preconsultation e-screen, there was a nonsignificant increase or no increase in consultation length; care providers felt that a slightly longer appointment was acceptable, given the increased disclosure of psychosocial issues [23-26,28]. Reviewing the results of e-screens helped care providers to plan consultations, set priorities, and engage with youth in useful discussions [19,22-24]. Adolescents believed that completing a screen by using a computer or mobile device afforded them increased privacy and confidentiality, which increased the likelihood of them disclosing psychosocial issues. In consultations, young people felt listened to, felt encouraged to talk, and felt that all of the issues that they wished to discuss had been addressed. Young people reported that the delivery and quality of brief interventions improved, and their satisfaction with care increased.

#### Acceptability and Feasibility

e-Screening for psychosocial concerns was found to be acceptable in 7 studies and was generally feasible to implement. However, all studies concluded that more research is needed into making e-screening for youth psychosocial issues feasible in primary care.

## Discussion

### Principal Results

More than one-third of adolescents engage in multiple risk behaviors [19,24]; therefore, the ability to conduct screening across several domains quickly and effectively in primary care might help with detecting issues that are not typically screened for by care providers. Multi-item e-screening tools for youth psychosocial issues have the potential to facilitate increased disclosure and, hence, early intervention in primary care settings. This review found 12 papers describing 11 studies that were carried out in a variety of settings in high-income countries. A range of study designs were used to evaluate the acceptability and feasibility of implementing e-screening tools for youth psychosocial issues in primary care settings. A lack of time is the most common barrier to screening among care providers; yet, when this was measured preconsultation, e-screening and subsequent discussion made little to no difference in consultation length [19,23-26,28]. The review of an e-screening report during ensuing discussions allows care providers to raise subjects that they may otherwise have found difficult to discuss. Despite concerns that young people may not want to address psychosocial issues in their consultations, youth participants reported increased satisfaction and felt more involved with their care when such discussions were initiated by their care providers. Additionally, reviewing e-screening results with young people directs discussions toward psychosocial issues and better meets the unique health and well-being needs of youth.

The reviewed studies found that while e-screening in primary care is effective in detecting youth psychosocial issues and enabling timely brief interventions, common barriers (a lack of time, training, tools, and staff and discomfort in raising sensitive issues) to their use persist [3,13,15,18,24,25,28,29]. The initiation of screens by a research assistant creates an artificial environment, and the initiation of screening does not become a part of daily workflows. Further, because some staff believe that e-screening requires extra resources, they may resist its integration into daily practice. The use of e-screening tools under research conditions does not represent their use within real, complex clinical environments, where context-specific barriers and challenges can inhibit the assimilation of e-screening tools into routine practice [30-39]. To overcome barriers to use, e-screening tools must be acceptable to intended users and involve usage processes that are feasible and can be easily assimilated into routine use [30,40-44]. Further, to reduce inequities among indigenous youth, screening tools also need to be culturally appropriate.

### Comparison With Prior Work

Existing evidence suggests that youth would prefer to complete an initial self-assessment electronically (e-screening) rather than undergo a face-to-face interview [21,45-47]. e-Screening not only saves staff time but also provides reliable and consistent results. Young people who believe that computers provide more privacy may disclose more sensitive issues in e-screens than they would in face-to-face interviews. Adolescents perceive e-screening as an appropriate method of collecting information in clinical settings [23,48-50]. Youth prefer to complete an e-screen in the waiting room prior to consultations with their clinicians [51]. This augments engagement with care providers, increases disclosure, and facilitates shared goal setting in ensuing consultations [52]. e-Screening for youth psychosocial issues in primary health care can improve health outcomes and help to reduce the incidence rates of youth suicide, self-harm, accidental death, and mental health issues [53].

For e-screening tools to be effective in improving patient outcomes, their use must become established in routine clinical practice. This challenges all clinic staff, individually and collectively, to make some degree of change in their ways of working and interacting with colleagues and patients.

The implementation of complex web-based interventions, such as e-screening, in particular clinical settings is influenced by how well these interventions are accepted, users’ perceptions of the benefits and barriers of these interventions’ uptake, and the impact that using these interventions has on the workflows of potential users and existing systems of practice [39].

As an implementation theory of action, the Normalization Process Theory [54] consists of 4 constructs (coherence, cognitive participation, collective action, and reflexive monitoring) that outline what intended users need to do to make sense of, commit to, engage with, and evaluate complex interventions [41,55-57]. The successful implementation of e-screening needs to begin by working in collaboration with stakeholders, community and cultural leaders, and end users, so that interventions are tailored to be acceptable and feasible for use in each specific setting. This co-design approach gives researchers a unique insight into the challenges faced by users in any given setting. Further, in a co-design approach, the experience, knowledge, and skills of users are used to inform the development of implementation processes and overcome context-specific barriers.

### Strengths and Limitations

This study’s strengths include the searching of 4 databases and a hand search, which were conducted to find studies for inclusion in this review. Explicit inclusion and exclusion criteria were used to identify the 12 studies that were finally reviewed, and to ensure that a comprehensive search was conducted, expansive search strings were developed. However, there are limitations to this review. There is a paucity of literature in this area, and most studies had considerable limitations to their methodologies and generalizability. Further, most of our findings pertained to only a subset of the reviewed studies. Finally, the heterogeneity of the studies included in this review precluded the ability to conduct a meta-analysis.

### Conclusion

The efficacy and acceptability of using e-screening tools are not in doubt. Nonetheless, their use in practice is sporadic and is often limited to youth who are considered to be at high risk [18,22]. The feasibility of implementing e-screening does not only rely on the availability of appropriate technological infrastructures; the effect that e-screening tools have on those who use them is also crucial to their efficacy [40]. When those who conduct screening recognize that there are clear benefits, such as improving the health outcomes of patients while reducing workloads, then the routine use of e-screening becomes viable [40,54]. To be truly effective, screening tools must be implemented in clinical settings, and their use must become a part of routine practice [30,40]. Co-designing and tailoring e-screening tools and processes to meet the needs of specific clinical contexts may be required to enable clinicians to overcome perceived barriers and integrate the use of e-screening processes into their practices.

## Appendix

Multimedia Appendix 1 Search strings.

## Abbreviations

HEEADSSS: Home, Education/Employment, Eating, Activities, Drugs and Alcohol, Sexuality, Suicide/Depression, and Safety
PRISMA: Preferred Reporting Items for Systematic Reviews and Meta-Analyses
