# Supplementary material for: Implementing the Routine Use of Electronic Mental Health Screening for Youth in Primary Care: Systematic Review
Source: JMIR Ment Health. 2021 Nov 19;8(11):e30479. doi: 10.2196/30479 (PMC8663603; doi:10.2196/30479)
Supplement: Multimedia Appendix 1 [file mental_v8i11e30479_app1.docx]

**Supplementary Table 1 Search strings**

| Search terms | Search string used |
| --- | --- |
| Screening | (screen* OR case-find*) |
| Youth | (youth OR adoles* OR teenager OR "young person" OR "young adult") |
| Mental health or risk behaviours | ("mental health" OR "risk* behav*" OR smoking OR tobacco OR nicotine OR "substance abuse" OR "alcohol*" OR "exposure to abuse" OR "eating disorder" OR sex* OR depression OR anxiety OR gambling OR anger* OR "conduct disorder" OR "physical inacti*" OR stress) |
